# Extracellular Vesicles in Cancer Drug Resistance: Implications on Melanoma Therapy

**DOI:** 10.3390/cancers15041074

**Published:** 2023-02-08

**Authors:** Alice Musi, Laura Bongiovanni

**Affiliations:** 1 Department of Veterinary Medicine, University of Teramo, 64100 Teramo, Italy; 2 Department of Biomolecular Health Sciences, Faculty of Veterinary Medicine, Utrecht University, 3584CT Utrecht, The Netherlands

**Keywords:** cancer, melanoma, extracellular vesicles, drug resistance

## Abstract

**Simple Summary:**

Melanoma represents only 1% of human skin cancers, but in several cases can lead to the death of the patient. Nowadays, there are different systemic therapies used for the treatment of human melanoma. Although these substantially improve patients’ lifespan, they are still associated with resistance. Extracellular vesicles (EVs), tiny vesicles released by tumor cells involved in intercellular communication, play an important role in melanoma pathogenesis and progression. They are crucially involved in several mechanisms of cancer drug resistance in several types of cancer, and there is a strong indication that EVs released by melanoma cells might play a role in the development of resistance, modulating the response towards anti-cancer drugs. Understanding their role will help improve the outcome of melanoma treatment.

**Abstract:**

Extracellular vesicles (EVs) are involved in the pathogenesis of neoplastic diseases. Their role in mediating drug resistance has been widely described in several types of cancers, including melanoma. EVs can mediate drug resistance through several different mechanisms, such as drug-sequestration, transfer of pro-survival proteins and RNA, induction of cancer stem cell-like features and interaction with cells of the tumor microenvironment and immune-system. Melanoma is a highly immunogenic tumor originating from the malignant transformation of melanocytes. Several therapeutic strategies currently used in the treatment of melanoma and the combination of BRAF and MEK-inhibitors, as well as immune check-point inhibitors (ICI), have consistently improved the overall survival time of melanoma patients. However, the development of resistance is one of the biggest problems leading to a poor clinical outcome, and EVs can contribute to this. EVs isolated from melanoma cells can contain “sequestered” chemotherapeutic drugs in order to eliminate them, or bioactive molecules (such as miRNA or proteins) that have been proven to play a crucial role in the transmission of resistance to sensitive neoplastic cells. This leads to the hypothesis that EVs could be considered as resistance-mediators in sensitive melanoma cells. These findings are a pivotal starting point for further investigations to better understand EVs’ role in drug resistance mechanisms and how to target them. The purpose of this review is to summarize knowledge about EVs in order to develop a deeper understanding of their underlying mechanisms. This could lead to the development of new therapeutic strategies able to bypass EV-mediated drug-resistance in melanoma, such as by the use of combination therapy, including EV release inhibitors.

## 1. Introduction

Extracellular vesicles (EVs) are particles which are naturally released from cells and are delimited by a lipid bilayer. They cannot replicate since they do not contain a functional nucleus [1]. EVs are produced and released by cells to communicate with each other and are internalized through fusion, phagocytosis or endocytosis, into the recipient cells where they exercise their function releasing their bioactive content (i.e., RNA, DNA, proteins, lipids) into the cytoplasm [2]. Anti-cancer drug resistance is a major challenge for advanced cancer treatment. An understanding of the underlying mechanisms and the development of effective strategies against anti-cancer drug resistance is desperately needed in the clinic and, therefore, represents some of the most explored fields in EV research [2].

The aim of this review is to summarize the EV-mediated mechanisms of resistance in tumors and to integrate and discuss the studies that have investigated their role in melanoma. Although only relatively few studies have been carried out, what has been observed until now suggests that EVs play a crucial role. The knowledge of these mechanisms represents the point of reference for a better understanding of the development of resistance to certain drugs by melanoma cells. This could provide new strategies to bypass this resistance and to obtain effective drug combinations for a successful treatment of human melanoma.

## 2. EVs: A Brief Introduction of Their Physiological and Pathological Roles

EVs can be classified, in terms of their biogenesis, as exosomes (produced within the multivesicular bodies in the cytoplasm and released by their fusion with the membrane) and microvesicles (released by budding of the cellular membrane). Similarly, in terms of their size, EVs are typically classified as small EVs (with a diameter ranging from 50 nm to 150 nm), the majority of which correspond with exosomes, and large EVs (from 100 nm to 1 mm) often corresponding with microvesicles. Apoptotic bodies (from 50 nm to 5 mm) are also a type of EV, produced and released by budding of the membrane of dying cells [2]. EVs can be found in various body fluids such as blood, saliva, breast milk and urine [3,4,5]. These can be collected with non- or minimally invasive methods, in the so-called liquid-biopsy. From liquid biopsies, EVs can be isolated with different methods to analyze their cargo. The level of purity of the final samples obtained can differ depending on the chosen isolating technique [6]. The methods most frequently used to isolate EVs are reported in Table 1.

Within the body, EVs play an important role from the earliest stages of embryonic life as they contribute to the implantation of the trophoblast [8], they prevent polyspermy [9] and facilitate sperm-egg fusion [10]. Beyond that, they continue to play a crucial role in maintaining tissue homeostasis, taking part in the regulation of several physiological processes. For instance, EVs can regulate blood pressure [11,12], promote angiogenesis and are involved in coagulation: those derived from platelets enhance the creation of new blood vessels and blood EVs can expose procoagulant tissue factor on their surface [13]. Their action has been studied in the renal system, where under healthy conditions they mediate intra-nephron communication and contribute to the maintenance of water and salt balance [14]. In the skeletal muscle, the role of EVs has also been investigated, showing that development and differentiation of the muscle after an intense exercise could also be EV-dependent [15]. Many studies focus on the role of EVs in the central nervous system, indicating their release from all the different cell types participating in neurotransmission. It has been demonstrated that EVs are essential for the maintenance and regeneration of synapses, as well as in axonal regeneration. Their levels increase with elevated neuronal activity and their cargo controls both phenotypic change and neuronal gene expression [2]. EVs also play a pivotal role in the regulation of the immune system. They contribute to the regulation of immune suppression preventing autoimmunity and chronic inflammation [14]. EVs actively participate in the immune response during inflammatory and infectious diseases, with an increase in patients’ circulating EVs released by neutrophils, monocytes and monocyte-derived macrophages [16,17]. Their participation is also essential for the initiation and resolution of inflammation [18].

During disease processes, EVs dramatically contribute to pathogenesis, playing a fundamental role in the development and progression of several types of diseases. Besides infectious and inflammatory processes, they are also involved in degenerative diseases and cancer. EVs and the specific functions of their cargo have been extensively studied in the pathogenesis of neoplastic disease. Being part of the tumor nano-environment (TNE), ref. [19] EVs are essential for intercellular communication, contributing to the formation of the tumor microenvironment (TME) thus, enhancing tumor growth and progression.

**Figure 1:**
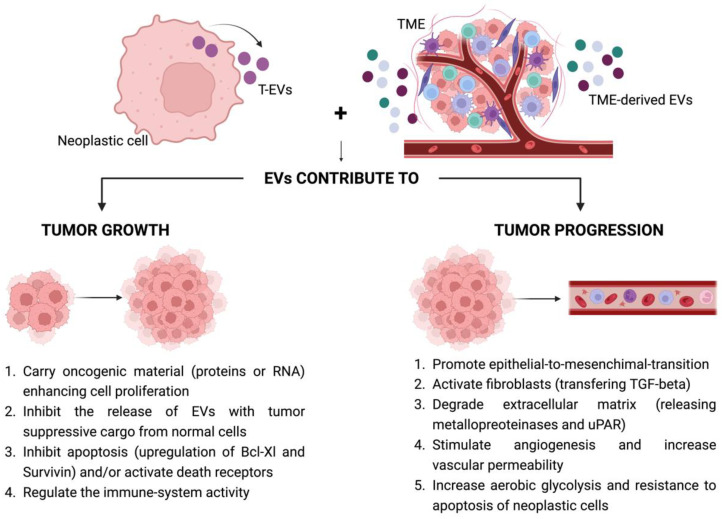
Mechanisms mediated by tumor-derived EVs and stromal cell-derived EVs that promote tumor growth and progression. EVs: extracellular vesicles; T-EVs: tumor-derived extracellular vesicles; TME: tumor microenvironment.

The main aim of the present review is to illustrate and discuss the importance that EVs can have in mediating drug resistance, particularly in melanoma treatment. In the literature, several reviews report how EVs contribute to tumor development, growth and progression; thus, we will not go in depth on this topic. However, since cancer-associated EVs and EVs derived from the cells of the TME play an important role in the reorganization of the cell metabolism [20], it is important to at least mention the leading mechanisms by which EVs can contribute to these processes (Figure 1). This can also be helpful to better understand how they and their cargo can interfere with anti-cancer drug mechanisms of action.

The ways by which tumor-derived EVs (T-EVs) are involved in tumor growth are numerous and include both the uptake of EVs carrying oncogenic material (such as RNA) by tumor cells and inhibiting the release from normal cells of EVs with tumor-suppressive cargo [21]. T-EVs can also regulate apoptosis; they can inhibit programmed cell death, for example, by upregulating the anti-apoptotic proteins Bcl-xL [22] and survivin [23] in recipient tumor cells. In addition, being enriched with FAS and TRAIL, they can activate lymphocyte death receptors, resulting in killing them [21].

T-EVs also have an immunomodulatory activity directly stimulating or inhibiting the immune system, regulating the activity of T cells and interfering with the complement cascade [21]. For instance, T-EVs carrying prostaglandin-E2 (PGE2) can suppress the activity of dendritic cells; T-EVs carrying TGF-β hinder the action and proliferation of natural killer cells; T-EVs can induce the polarization of tumor-associated macrophages (TAMs) towards a typically pro-malignant M2-phenotype [24]. Tumor progression is a process determined by a large number of events, also involving cells of the TME. T-EVs participate in this process through different mechanisms, such as: (a) promoting epithelial-to-mesenchymal transition; (b) activating fibroblasts (in cancer associated fibroblasts, CAFs) by the transport and transfer of TGF-β [25,26,27,28], resulting in extracellular matrix degradation and induction of cancer-promoting cytokines [2]; (c) releasing metalloproteinases [2] or uPAR (urokinase-type plasminogen activator receptor) [29,30,31] that degrade the extracellular matrix and the basement membranes; (d) inducing angiogenesis (i.e., transferring to endothelial cells molecules increasing glycolysis [20]), which helps to overcome oxygen and nutrition deficiency by activating endothelial cells to stimulate vascularization [32]; and (e) increasing the permeability of endothelial cells [21], down-regulating proteins linked to cell-junction activity (i.e., α/β-catenin, E/N-calmodulin) [33,34,35,36].

Furthermore, it must be considered that not only T-EVs play an important role in tumor growth and progression. Additionally, cells of the TME can release EVs (stromal-derived EVs) able to reprogram cell metabolism both of cancerous and stromal cells [20]. For instance, M2-macrophages can increase aerobic glycolysis and resistance to apoptosis in breast cancer cells by transferring HISLA (HIF-1α-stabilizing lncRNA) [37]; EVs released from CAFs contain several molecules and metabolites that enhance tumor cell proliferation in prostate cancer [38]; EVs derived from mesenchymal stem cells can upregulate the PI3K/AKT pathway and HIF-1α and downregulate the protein GLUT1, promoting the growth of osteosarcoma [39]; EVs derived from adipocyte cells can carry and transfer protein involved in the fatty acid oxidation (FAO) process, enhancing migration and invasion in melanoma and prostate cancer cells [40,41] (Figure 1).

In melanoma, the role of EVs in growth and progression has been demonstrated by numerous research works. In a recently published review, it was reported that the progression and development of metastasis in melanoma also depends on EVs, and in patients with different stages of melanoma, EVs have shown different cargo [42]. For example, higher levels of CD63, S100B, MIA and PD-L1 have been found in EVs released in the plasma of advanced melanoma patients compared with healthy controls [43,44,45,46]. Melanosomes, a particular type of EVs specifically released by melanoma cells, are able to induce phenotypic changes in dermal fibroblasts by carrying miR-211, ascribing them features of CAFs already at the early stage of the disease [47]. Interestingly, in patients with melanoma, a large number of EVs are found in the blood and the lymphatic system. These EVs are able to reach lymph nodes and contribute to the creation of a premetastatic environment, creating a favorable condition for neoplastic cells and enhancing the formation of metastasis [2,48].

Overall, EVs play a key role in melanoma development and progression, carrying different bioactive molecules with differing functions depending on their releasing cell and the progression of the disease. Furthermore, the fact that EVs can be obtained with non-/minimally invasive methods from different biofluids renders them the ideal candidate to develop new, reliable and useful biomarkers to diagnose and monitor the disease and therapeutic effectiveness, as well as to glean valuable information about prognosis. Numerous published studies have investigated the role of EVs in melanoma development, progression, diagnosis and treatment in recent years. These have been reviewed in several recently published reviews, including Moosavian et al. (2022) Lattmann and Levesque (2022) [49,50]. Other reviews specifically focus on the role of EVs in the modulation of the melanoma-associated immune response and immunotherapy [51,52].

Our hypothesis is that having a deeper understanding of the roles of EVs during melanoma progression and treatment could also lead to an improvement of the effects of currently available treatments limited by the still high rate of treatment failure.

## 3. EVs Implication in Cancer-Drug Resistance

Anti-cancer drug resistance is a major challenge for advanced cancer treatment. An understanding of the underlying mechanisms and the development of effective strategies against anti-cancer drug resistance are desperately needed in the clinic and represent some of the most explored fields in EV research. 

Anti-cancer drug resistance is linked to several mechanisms associated with both irreversible genetic mutations and reversible proteomic and epigenetic mechanisms [53]. 

**Figure 2:**
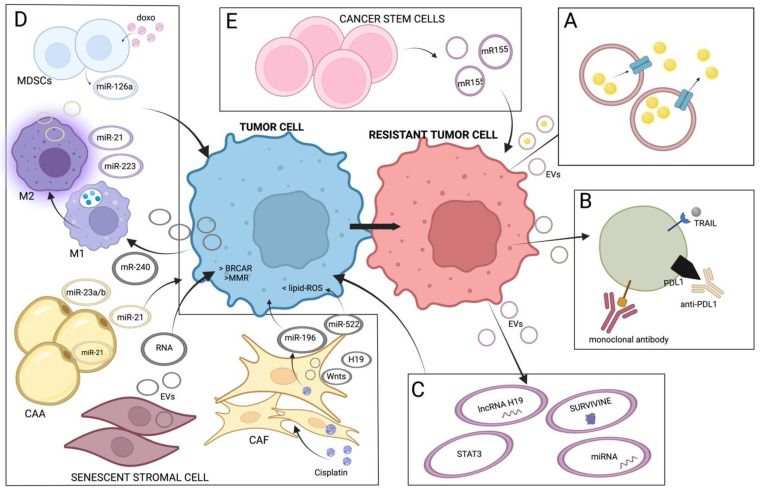
Drug resistance can be mediated by extracellular vesicles (EVs) through different mechanisms: (**A**) drug sequestration and drug efflux; (**B**) surface exposure of anti-drug factors; (**C**) transfer of resistance molecules from resistant to sensitive tumor cells after drug exposure or stress or suffering conditions; (**D**) transfer molecules that enhance resistance between the cells of the tumor microenvironment and the neoplastic cells; (**E**) EVs released from cancer stem cells: induction of cancer stem cell-like features. EVs: extracellular vesicles; CAF: cancer associated fibroblasts; CAA: cancer associated adipocytes; M1: M1-type macrophages; M2: M2-type macrophages; MDSCs: myeloid-derived suppressor cells; Doxo: doxorubicin.

Furthermore, tumor heterogeneity and microenvironment-mediated mechanisms have gained increasing attention, since it was demonstrated that they contribute significantly to the capacity of tumor cells to escape therapeutic effects [53]. However, the way by which resistance arises in cancer cells is still not fully understood. In this context, the role of EVs has emerged as a key contributor. Under the effect of a specific antitumor compounds, both sensitive (dying cells, senescent cells) and resistant tumor cells (such as cancer stem cells), as well as non-tumor cells of the microenvironment, will start to produce more and different EVs. The altered cargo present in drug-induced EVs appears to participate dynamically in the drug resistance mechanisms related to the use of chemotherapy, targeted therapy and immunotherapy, through several different action mechanisms (Figure 2). However, most of the functions of EV-derived molecules in the development of anti-cancer drug resistance remain to be discovered [21].

In the following paragraphs and in the schematic illustration of Figure 2, we summarize the main mechanisms of EV-mediated drug resistance that have been reported, including some examples of studies performed in tumor types other than melanoma, to provide a more complete picture.

A. Drug-sequestration and drug-efflux

Shedden et al. first reported the ability of EVs to accumulate and expel drugs in 60 different tumor cell lines, demonstrating the accumulation of doxorubicin as well as other molecules in the released vesicles [54]. Drug efflux through the plasma membrane is normally regulated by components of the ATP-binding protein family, and its three most important members are ABCB1, ABCC1 and ABCG2 [55]. It has been demonstrated that some EVs secreted by tumor cells can contain ABCB1, being therefore able to transfer the capacity of carrying away the drug from recipient, sensitive cells [56].

B. Surface exposure of anti-drug factors

EVs are also capable of exposing specific antigens/ligands or receptors on their surface that can bind anti-cancer antibodies or directly recognize anti-cancer compounds, in order to remove or counter the effects of these molecules from the extracellular space [57]. We report some examples below:

- EVs released from glioblastoma cells contain high levels of PD-L1 and are able to capture the immunotherapeutic antigen, reducing its effect [58].
- Moreover, EVs coated with DR5 (death-receptor 5) can indeed reduce the sensitivity of colon cancer cells to TRAIL by the sequestration of the pro-apoptotic ligand, thus limiting the sensitivity of these cancer cells to the drug [59].
- Resistance to therapeutic monoclonal antibodies used in specific tumor types, such as B-cell lymphoma and breast cancer, has also been linked to EV-mediated transport of resistant factors. B-cell lymphoma cells secrete EVs containing CD20 and can protect lymphoma cells from antibody attack by binding the therapeutic anti-CD20 antibodies [60].
- In a similar mechanism of action, breast cancer overexpressing human epidermal growth factor receptor HER2 treated with trastuzumab (anti-HER2 antibody) can inhibit its effect by secreting EVs enriched with the same receptors [61].

C. Transfer of resistance molecules from resistant to sensitive tumor cells after drug exposure or stress and suffering conditions (i.e., hypoxia)

Several studies have pointed out the differences in the cargo composition between EVs produced by drug-resistant and drug-sensitive cancer cells, identifying numerous deregulated miRNA (small, around 22 nucleotides-molecules of endogenous and non-coding RNA) and/or proteins that might play a role in therapy resistance [62,63,64]. A few of them also confirmed the functional role of these EVs in changing the behavior of the recipient, sensitive tumor cells.

Regarding the EV-carried proteins, several studies tried to understand their underlying mechanism of action:

- EVs released from prostate and cervical cancer contain a higher amount of survivin (a protein that can inhibit apoptosis acting as a marker in several types of cancers) with a limitation of genetic damages, since survivin protects cells from genotoxic stresses and proton irradiation [65,66,67]. Kreger et al. discovered that breast cell line MDAMB231 treated with paclitaxel (PTX, a chemotherapeutic drug that acts through the stabilization of microtubules) releases exosomes (a specific subset of EVs) enriched with survivin and that these exosomes were able to confer resistance. They then incubated the SKBR3 breast cancer cell line with exosomes derived from DMSO-treated, as a control, and PTX-treated MDAMB231 cell line. After the treatment of the SKBR3 cell line with the PTX, they observed a variation in the sensitivity when survivin-enriched exosomes were added [68].
- Ovarian cancer cells cultured under hypoxic conditions can develop resistance toward cisplatin due to hypoxia-induced EVs by the spreading of the transcription factor STAT3 [69]. EVs produced under hypoxic conditions are enriched in STAT3, which acts as an oncogenic factor. Under hypoxic conditions, the amount of STAT3 increases and, by regulating Rab7 and Rab27, leads to the release of a higher number of EVs. Adding STAT3-inhibitors on ovarian cancer cells and treating them with cisplatin increases their sensitivity, leading to the hypothesis that hypoxic ovarian cancer cell-derived exosomes play an important role in the development of resistance toward this drug [69].
- EVs can also activate the PI3K/AKT pathway, which is involved in the progression of most neoplasias and in the proliferation of cancer cells [70]. It has been demonstrated that small EVs released from invasive hepatocellular carcinoma (HCC) cell lines promote sorafenib resistance in hepatoma cells in vitro through the activation of the HGF/c-Met/Akt signaling pathway and through the inhibition of sorafenib-mediated apoptosis; moreover, authors showed that resistance was not only observed in vitro, but also in vivo [71].

The role of different types of EV-derived RNA has also been extensively investigated and proved:

- MiRNA are released from cancer cells via EVs and, once they are internalized by sensitive cells, mediate drug-resistance. Different types of miRNA can increase the resistance of several types of cancer [57]. Varying biological processes, such as cell proliferation, cell growth and apoptosis, are regulated by the PTEN/PI3K/AKT signaling pathway. PTEN is the product of PI3K, and a reduction in its activity has been observed both in primary and metastatic cancers (i.e., following methylation) [72]. It has been discovered that this pathway represents the target for different EV-miRNAs related to drug-resistance [55]: (a) miR-32-5p has been found in EVs released from 5-fluorouracil (5-FU)-resistant cells of hepatocellular carcinoma, reducing the expression of PTEN, thus enhancing multidrug resistance [73], (b) miR-21 has been detected in EVs released from cisplatin-resistant cells of an oral squamous cell carcinoma and it can be transferred to sensitive cells, increasing their resistance. In addition, this miRNA causes the decrease in PTEN [74].
- Additionally, lncRNA, long non-coding RNA, with more than 200 nucleotides, are capable of transferring the ability to survive different anticancer drugs to several cancer cells [57]. One of the most studied lncRNAs is H19. It can be found in small EVs released from non-small cell lung cancer cells. The uptake of these EVs from the recipient cells leads to the downregulation of miR-615-3p and the upregulation of ATG7 (i.e., a regulator of autophagy whose increase leads to erlotinib resistance) [75]. H19 has also been found in EVs released from tumor stromal cells (CAF) during colorectal cancer, enhancing oxaliplatin resistance both in vivo and in vitro [76].

D. Transfer of molecules that enhance resistance between the cells of the TME and the neoplastic cells.

Tumor tissue is made up both of tumor cells and normal cells of the tumor microenvironment (TME), in which the cells communicate with each other. As stated above, cells of the TME, (e.g., fibroblasts, adipocytes, endothelial cells, cells of the immune system), are activated and modified in their behavior by tumor cells and are key factors in cancer progression [57]. It has been demonstrated that the cells of the TME also have an important role in the development of drug resistance through the release of EVs [77]. Indeed, a bi-directional communication between cancer cells and stroma cells through EVs has been observed during the exposure to anti-tumor drugs. There are several published articles describing this active communication via EVs in several types of tumor, and some examples are presented below:

- It has been observed that senescent stromal cells can generate genomic alterations in leukemic/lymphoma cells through the transfer of EVs containing specific miRNAs that modulated BRCA1 and MMR (mismatch repair system, for genome stability) pathways, rendering them resistant to chemotherapy [64]. EVs derived from stromal cells can also activate several pathways (i.e., JNK, p38 and Akt) leading to an increased resistance towards bortezomib [78].
- Myeloid-Derived Suppressor Cells (MDSC) can also control the efficacy of some chemotherapeutic drugs [79]. Doxorubicin-induced MDSCs can release miR-126a-enriched EVs able to enhance the resistance of breast cancer cells towards doxorubicin [80].
- Under the influence of chemotherapeutic drugs such as oxaliplatin or 5-fluorouracil, non-tumor cells of the TME, such as CAFs and CAAs (cancer-associated adipocytes), refs. [62,64,81] can also release EVs, influencing, in turn, tumor cell behavior. Notably, it has been demonstrated that CAFs are innately resistant to some specific drugs such as cisplatin. Small EVs from CAFs exposed to cisplatin can confer chemoresistance and an aggressive phenotype in head and neck cancer cells through the transfer of EVs enriched with functional miR-196a [62]. CAF-derived EVs can also carry other miRNA, lncRNA and proteins that confer resistance to the recipient cells. For example, in gastric cancer cells, miR-522-enriched CAF-EVs limit the accumulation of lipid-ROS, decreasing the efficacy of chemotherapy [82]. Additionally, H19-enriched and Wnts-enriched CAF-EVs promote chemoresistance in colorectal cancer [76,83,84]. CAAs, having a higher level of miR-21 than ovarian carcinoma cells, are able to transfer it to tumor cells and induce chemoresistance. Moreover, with the reduction in the EV-mediated transfer of miR-21, cells regain sensitivity to the drug [81,85]. Similarly, it has been observed that CAA-derived EVs can transfer miR-23a/b to hepatocellular carcinoma cells, activating the VHL/Hif axis and increasing 5-FU (5-fluorouracil) resistance [86].
- The connection between cancer cells and immune cells of the TME can also be regulated by EVs and their cargo. The main immune cell population that can be found in the TME is the macrophagic one. Generally, M1-type macrophages are commonly associated with a better prognosis. Conversely, M2-type is associated with cancer progression, promotion of angiogenesis and remodeling of the tumor extracellular matrix, resulting in the development of drug resistance [87]. Chen et al. demonstrated that EVs enriched with miR-940 can lead to M2-type polarization [88]. Furthermore, macrophages can deliver, via EVs, miR-21 to gastric cancer cells [89] and miR-223 to ovarian carcinoma cells to gain a chemo-resistant phenotype [90].

E. EVs released from intrinsically resistant cancer stem cells: induction of cancer stem cell-like features.

Cancer stem cells (CSCs), which are intrinsically quiescent and slow-cycling, tend to be resistant to chemotherapy [91,92]. Under the effect of drugs, they can produce EVs that can modify normal surrounding cells to promote immune tumor escape, tumor growth and metastasis, and also confer drug resistance to neighboring drug-sensitive cancer cells. This was demonstrated, for example, in a breast cancer model where CSC-derived EVs enriched with miR-155 were responsible for the acquisition of chemoresistance in recipient breast cancer cells [64]. Different studies have demonstrated that EVs released by CSCs in different tumor types have a role in tumor progression and drug resistance, as thoroughly reviewed by Lindoso et al. (2017) and Zhang et al. (2017) [93,94]. However, many aspects still need to be addressed and understood. A better characterization of CSC-released EVs and their role in the crosstalk between CSCs and their stem cell niche will open the way to new therapeutic strategies contributing substantially to the improvement of currently used therapies.

## 4. EVs Mediate Drug Resistance in Melanoma

### 4.1. Systemic Therapies for Melanoma

According to the American Cancer Society, melanoma represents only 1% of skin cancers in humans, but in several cases it can lead to the death of the patients. The first-line defense for the treatment of this neoplasia is surgical resection. It has been demonstrated that resection of melanoma is associated with a decreased number of circulating EVs in humans [42,95]. 

Nowadays there are different systemic therapies used for the treatment of human melanoma, such as neoadjuvant/adjuvant therapy or primary treatment:

- Chemotherapy: cisplatin, temozolomide, vincristine and vinblastine are some of the chemotherapeutic drugs most frequently used to treat advanced-stage melanoma [96]. This happened in the past when this was the only systemic therapy used to treat stage IV melanoma patients. Nowadays, even if a reduction in tumor size occurs with the administration of these drugs, there is no evidence of survival advantages [97]. Currently, chemotherapy is almost no longer administrated in most melanoma patients and represents only a last-line treatment in those cases in which a resistance towards ICI and target therapy occurs [98]. However, chemotherapy can still be used when it represents the only alternative available and for those melanoma patients without BRAF mutation who develop a toxicity reaction after the administration of ICI [97];
- Small Molecule Targeted Therapy: such as BRAF-inhibitors (i.e., vemurafenib, dabrafenib), MEK-inhibitors (i.e., trametinib) and the combination of BRAF and MEK-inhibitors [96]. About half of cutaneous melanoma patients carry BRAF V600 mutation. An increase in the overall survival and progression-free survival occurs after the administration of vemurafenib and dabrafenib in patients with BRAFV600E mutation [99,100]. Additionally, an increase in the response rate, in the overall survival and in the progression free survival, occurs after the administration of BRAF and MEK-inhibitors in combination [97].
- Immune Checkpoint Inhibitors (ICI): such as CTLA-4 inhibitors (i.e., ipilimumab) and anti-PD1 therapy (i.e., nivolumab, pembrolizumab). The combination of nivolumab and ipilimumab leads to the best outcome, and the Food and Drug Administration (FDA) approved this cocktail as the best treatment for both advanced BRAF-negative melanoma patients [96,101,102] and for melanoma patients with BRAF mutation [97]. Up to now, since PD-1 antibodies have been approved, ipilimumab is used in combination with the antibodies or by itself as a second treatment option [97].

The scientific community is trying to both improve the effectiveness of these melanoma therapies by limiting their side effects and identifying the best candidate patients for each different type of therapy [97]. Considering the key role of EVs in melanoma pathogenesis and progression, and in light of their crucial involvement in several mechanisms of cancer drug resistance, there is a strong indication that EVs released by melanoma cells might play a role in the development of resistance, modulating the response towards anti-cancer drugs.

**Figure 3:**
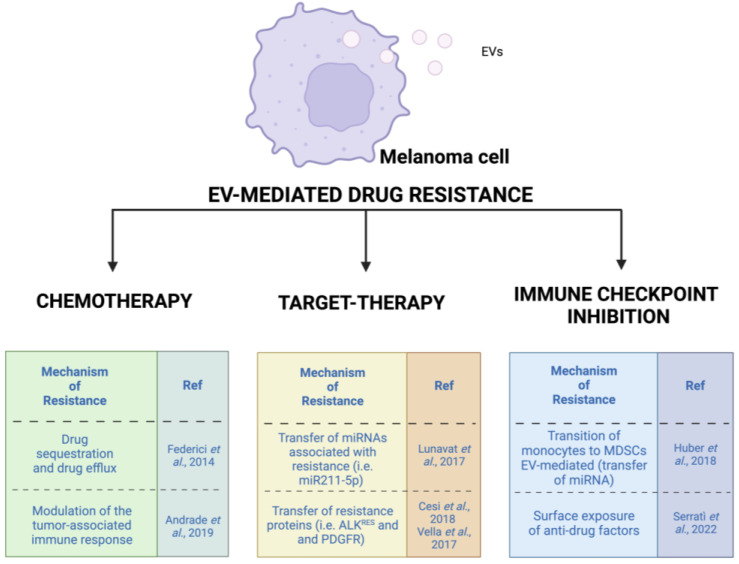
Described mechanisms of drug resistance involving EVs based on the main therapeutic approaches for melanoma [106,113,115,116,120,125,130].

The results of the most relevant published works on this topic, divided into different types of melanoma treatments, will be listed and discussed in the following paragraphs. However, to better understand the role of EVs in melanoma drug resistance, further studies are needed (Figure 3).

### 4.2. Mechanisms of EV-Mediated Resistance towards Chemotherapeutic Drugs

As mentioned above, chemotherapy currently represent only a last-line treatment, although it may rarely still be administrated [98]. Therefore, it is relevant to mention the published works investigating the EV-mediated mechanisms of resistance to these systemic drugs, which may be worthy of further investigation. 

It is well known that chemotherapy drugs are associated with resistance [103,104,105], and an increased number of EVs released by melanoma cells has been reported as a consequence of chemotherapy [106].

Cisplatin is an alkylating agent able to interfere with the DNA replication once inside tumor cells. The uptake and the efflux of cisplatin is regulated by several mechanisms and one of them is the pH variation. An acidic environment leads to the selection of cells with elevated levels of proton-pump activity, actively eliminating the drug and becoming resistant cells. The acidic environment itself contributes by lowering the extracellular pH, decreasing the pH inside EVs [107,108] and increasing the secretion of EVs from human melanoma cells. On the contrary, the administration of proton pump inhibitors (PPI) (anti-acidic drugs used for the inhibition of the H^+^K^+^ATPases) could reduce the release of EVs from tumor cells [109]. Hence, the hypothesis that acidic vesicles can play a fundamental role in the resistance towards cytotoxic drugs through both the sequestration and neutralization of alkaline drugs (i.e., cisplatin), as well as through the elimination of these molecules towards a vesicles-mediated mechanism [110,111,112].

Federici et al., in 2014, tried to better understand the process that leads human melanoma cells to the development of resistance towards cisplatin through exposing human melanoma cell lines to the drug. Results confirmed not only the important role played by acidification in cisplatin uptake by melanoma cells, but also highlighted the key role of EVs in the development of resistance. By the analysis of the content of EVs collected from the conditioned culture medium, cisplatin was found at differing levels depending on the pH of the medium. Higher levels of cisplatin were found in association with lower pH condition. Thus, their results strongly indicated the involvement of EVs in the secretory pathway leading to the elimination of the cisplatin and contributing to the development of drug resistance [113].

Drug sequestration and efflux are not the only methods used by EVs to decrease the response of melanoma cells to chemotherapeutic agents. It has been observed that in both human melanoma cell lines treated with temozolomide (TMZ) and in a murine melanoma cell line treated with cisplatin, alkylating drugs can induce the increase in EVs’ shedding and uptake by melanoma cell lines in a dose-dependent manner. In this case, however, the authors found that the EV-mediated resistance was not “direct”, through a melanoma cell-to-cell communication via EVs. In fact, no alteration in the sensitivity to TMZ or cisplatin was observed after the treatment of naïve melanoma cell lines with EVs isolated from the same cell line pre-treated with the drugs [106]. However, when incubating macrophages obtained from bone marrow cells with EVs isolated from melanoma cell lines pre-treated with TMZ, they noticed a change in the polarization toward a M2 phenotype, with an upregulation of M2-marker genes. Since the M2 phenotype is commonly associated with the development and progression of the tumor, this suggests that EVs can contribute to melanoma progression and to the development of resistance towards these drugs also through a different, “indirect” mechanism of action [106,114] by the modulation of the tumor associated immune response.

### 4.3. EVs Cargo in the Regulation of Target-Therapies

Around 50% of the patients with melanoma have a mutation in the Ser/Thr-Kinase BRAF (most of the time V600E). Therefore, the introduction of specific BRAF-inhibitors had represented a crucial point in the treatment of this neoplasia. However, the development of resistance with the reactivation of the MAPK pathway occurs quickly, highlighting the importance of focusing research on the mechanisms underlying this resistance [115]. 

Three studies that have specifically investigated EV-mediated drug resistance in melanoma towards BRAF-inhibitor therapy are discussed below. 

Two of them demonstrated that, after treatment with two commonly used BRAF inhibitors (vemurafenib and dabrafenib), the EVs released by resistant melanoma cells contained factors linked to melanoma resistance and BRAF inhibitors, such as miR211-5p and PDGFR. 

In the first of these two studies, Lunavat et al. (2017) observed that treatment with BRAF-inhibitors was able to change the miRNA cargo of EVs. They treated two melanoma cell lines with V^600E^ mutation (MML-1 and A375) and a primary melanoma cell line obtained from MML-1 cells transplanted mice, inducing an increased levels of miR-211-5p in both cells and small EVs isolated from their conditioned medium [116]. It is demonstrated that miR211-5p is involved in the resistance to BRAF-inhibitors, since RNA-sequence data have shown higher levels of this miRNA in resistant cells compared to sensitive cells [117,118]. Moreover, Lunavat et al. observed that melanoma cells transfected with mRNA-211-5p showed a reduced sensitivity to vemurafenib treatment and that the inhibition of mRNA-211-5p in the resistant cell line affected proliferation negatively. However, it needs to be further investigated whether EVs are indeed capable of transferring this active miRNA to sensitive cells by performing more mechanistic studies. 

Vella et. al. (2017) investigated whether EVs released by melanoma cells resistant to BRAF-inhibitors can modify the response of sensitive recipient cells after their exposure to these drugs. They used two melanoma cell lines: sensitive (LM-MEL-64) and resistant (LM-MEL-64R3). In the latter, resistance was due to the reactivation of the PI3K/AKT/AKT pathway, specifically linked to the higher phosphorylation of two RTKs (i.e., Receptors Tyrosine-Kinase), EGFR and PDGFRβ. In melanoma, after the exposure to BRAF-inhibitors, two different pathways can be reactivated, resulting in the development of resistance: the MAPK and the PI3K/AKT/AKT pathways [119]. With these experiments, the authors found that small EVs released from resistant melanoma cells had more PDGFRβ expression compared to sensitive cells and proved that EV-derived PDGFRβ can also be transferred from resistant to sensitive cell lines. They showed that when LM-MEL-64 exposed to LM-MEL-64R3 derived EVs were treated with PLX4720, the treatment did not reduce their growth compared to the ones treated with EVs from the sensitive cell line. Moreover, they demonstrated the reactivation, in the sensitive recipient cells, of the PI3K/AKT pathway in a dose-dependent manner; the more EVs the cells received, the higher the increase in pAKT detected. Authors also found that the development of resistance obtained through EVs carrying PDGFRβ was not just a prerogative of the cell line LM-MEL-64, but also of another melanoma cell line (M229AR) after the exposure to BRAF-inhibitors and linked to resistance [120]. All together, these results show that the role of PDGFRβ-carrying EVs is essential to preserving the functionality of one of the main mechanisms related to resistance after administration of MAPK-pathway inhibitors, the reactivation of the PI3K/AKT pathway.

In the last paper, Cesi et al. (2018) focused their attention on a truncated form of the receptor ALK (anaplastic lymphoma kinase), which they named ALK^RES^, upregulated in several neoplasia, including melanoma. Eleven % of melanoma tissues present a truncated ALK transcript resulting in a smaller protein, which was shown to be oncogenic [121]. ALK^RES^ is involved in the development of BRAF-inhibitor resistance through the reactivation of MAPK pathways, while in the absence of the mutation, resistant cells respond to both BRAF and MEK inhibitors. Using an in vivo assay in A375-X1-resistant melanoma cell-transplanted mice, they first proved that treatment with combined BRAF and ALK inhibitors can stop tumor growth. Subsequently, they demonstrated that this resistance can be transmitted through EVs. EVs can transfer ALK^RES^, and by proteomic analysis of the EV cargo they also demonstrated that ALK^RES^ remain functional after being transferred in recipient cells. Indeed, by the determination of the dose response to the BRAF-inhibitor PLX4032 following EVs’ uptake by IC_50_ calculation, they found no significant differences after incubation with sensitive-cells-derived EVs, while significantly higher IC_50_ was measured after incubation with resistance-cells-derived EVs, demonstrating that the drug resistant phenotype can be mediated by EVs [115].

These three studies represent a fundamental starting point to further understand the role of EVs in target-therapy resistance mechanisms in melanoma and they prove the fundamental role of EVs in the development of melanoma-resistance regarding the use of BRAF-inhibitors that are worth further investigations.

### 4.4. The Role of EVs in ICI

The immunogenicity of a tumor is its ability to activate an adaptive immune response able to prevent its growth. However, the fundamental prerogative is that cells must express an adequate amount of antigen capable of raising immune activation instead of immune tolerance [122]. Melanoma is one of the most immunogenic tumors, and there are frequently many immune cells reflecting a reaction of the host towards the neoplasm. However, tumor progression can also occur in the presence of an antitumor response implemented by the immune system; melanoma is able to progress in the presence of an abundant lymphocytic infiltrate, suggesting that the immune response can fail in effectively controlling tumor growth. Human melanoma often develops in an environment rich in immune cells, especially lymphocytes, which release their cytokines, contributing to an anti-tumor response [123]. Melanoma cells have a unique immunogenic profile and provide a model for investigating the molecular interaction of neoplastic cells with those of the immune system. Several studies in this field contributed to the discovery of new target molecules on immune cells for the development of effective therapeutic strategies [122]. Several studies have been performed discovering new biomarkers (in liquid biopsies) able to predict response to treatment to ICI and a number of molecules (protein and RNA) have been identified [124,125]. 

The role of EVs in immune response has been extensively studied, including their role in melanoma development and progression [51,52]. By analysis of the literature, several studies can be found demonstrating the utility of EVs as predictive factors in melanoma: PD-1^+^ [124,125], PD-L1^+^ [125], CD8^+^ [124] and uPAR^+^ [126] EVs represent biomarkers able to predict the response to ICI. However, only a few studies tried to deeply investigate the mechanism through which EVs can contribute to the development of resistance towards ICI. Myeloid-derived suppressor cells (MDSCs) are immature myeloid cells whose presence is associated with cancers. They can inhibit the activity of antitumor T cells [127] and are able to activate pathways associated with cell resistance [128,129]. Huber et al. showed by in vitro studies that melanoma EVs can mediate the transition of monocytes to MDSCs [130]. Incubating CD14^+^ monocytes for 24 h with EVs isolated from melanoma cell cultures, authors observed the differentiation of the cells towards a new phenotype that they called EV-MDSCs. These cells were able to downregulate the expression of HLA-DRA (Major Histocompatibility Complex, Class II, DR Alpha), enhance the transcription of IL-6 and CCL2 and inhibit the activity of T cells. Similar results were obtained incubating CD14^+^ monocytes for 24 h with EVs isolated from the plasma of patients with advanced melanoma [130]. Further investigating the underlying molecular mechanisms by genome-wide transcriptional analyses, the authors showed that under the phenotypic change of CD14+ cells, the transfer of miRNAs occurred. Since the lack of local T cells’ immune reactivity is one of the causes of resistance towards ICI, they demonstrated that EVs can contribute to the development of resistance, also mediating this phenotypic change of CD14+ cells. Furthermore, Xiao et al. showed that EVs released from melanoma cells bring two different types of miRNAs (miR-191 and Let-7a) that can modulate the process, leading to: (a) melanoma phenotyping switching (a process similar to the epithelial-to-mesenchymal-transition, EMT), (b) loss of adhesion factors and (c) regression toward a mesenchymal phenotype [131].

Melanoma-derived EVs express large amounts of PD-L1 on their surface [132] and these EVs are able to reach the lymph nodes inactivating the T cell response [133]. Serratí et al., besides investigating the role of PD-L1^+^ and PD1^+^ melanoma EVs, also tried to understand their involvement in the development of resistance toward ICI [125]. They isolated EVs from the blood of responders and non-responder melanoma patients, stained them and then incubated with PBMCs previously isolated from the blood of healthy donors, responders and non-responders. Using LND1 cells (a BRAF wt melanoma cell line) they created spheroids, adding PBMCs onto them to study the trafficking towards the tumor of these cells. They observed a reduction in the trafficking to the tumors for the PBMCs derived from melanoma patients compared with those obtained from healthy donors. Moreover, they showed a decrease in trafficking for the PBMCs that had been previously incubated with EVs from responders and, above all, for those cells incubated with EVs from non-responders. Interestingly, after the addition of nivolumab on spheroids, cell death was reduced for those cells previously incubated with EVs from responders and, once again, the phenomenon was even more evident for those incubated with Evs from non-responders [125]. 

Taken together, these results represent the proof that EVs are directly involved in the development of resistance in ICI-therapy.

## 5. EVs Inhibitors as a Strategy to Bypass Cancer Drug-Resistance

EVs represent a highly heterogeneous population and several subpopulations of EVs involved in different processes can be characterized. Being able to discriminate these differences represents an essential prerequisite to subsequently acting on those EVs specifically involved in the development of resistance. The inhibition of the synthesis and/or the release of EVs represents a first strategy to increase therapeutic efficacy [134,135]. 

EV inhibitors can be divided into two main groups: those acting on the trafficking of EVs (i.e., Calpeptin, Manumycin A, Y27632) and those acting on lipidic metabolism (i.e., D-Pantethine; Imipramine; GW4869) [135]. Each compound acts through different mechanisms and the biological targets differs for each of them. Their roles have been investigated in vitro in several cell lines, including cancer cells, demonstrating that the treatment with EVs inhibitors cause a higher efficacy of the therapy [135]. For instance, Jorfi et al. found a larger amount of anticancer drugs inside the cells of a prostate cancer cell line treated with calpeptin in addition to docetaxel and methotrexate, compared to the same cell line treated with only the anticancer drugs [136]. Another study conducted in vitro on ovarian cancer cells treated with cisplatin demonstrated that adding EVs uptake-inhibitors could be a way to sensitize the cells to the drug, improving the efficacy of the treatment [137]. This has been possible thanks to previous studies that showed how EVs are involved in the development of resistance in this type of cancer. Another example of possible actions on EVs is shown in the study of Richards et al., in which the incubation of CAFs (previously treated with gemcitabine) with an exosome-release inhibitor (GW4869) led to a better efficacy and response towards the drug on pancreatic cancer cells [138]. The efficacy of EVs inhibitors has been validated, also in vivo, where their use has been associated to a reduction in tumor growth [135]. In the aforementioned study of Jorfi et al., the consequence of the increase in the amount of the drugs at the intracellular level was observed in vivo, where authors described a reduction in tumor growth after the administration of the EVs inhibitor [136]. 

These are only a few examples that show how both the many in vitro studies, as well as the less numerous in vivo experiments, led to the hypothesis that EVs inhibitors could play an important role as adjuvants in cancer therapy. However, it should be taken into consideration that their use is not without side effects and that, as suggested by the Community of Extracellular Vesicles, there is still much to be done to determine non-toxic concentrations of these inhibitors as single or combined agents [135].

## 6. Considerations and Future Perspectives to Overcome Drug Resistance in Melanoma

This review was set up with the aim of assessing the importance of EVs in the development of resistance in several types of cancers, including melanoma. To develop a full picture of the role of EVs in mediating drug-resistance in melanoma and to unravel the underlying mechanisms through which this resistance is conferred, additional studies and future investigations are needed in order to set out in vivo and functional studies to confirm the in vitro results. The acquisition of this knowledge is essential to develop new therapeutic strategies that aim to counteract or at least attenuate EV-mediated drug resistance. A first strategy could be the prevention of EVs’ communication inhibiting the release, the transfer and/or the uptake of EVs. Matsumoto et al. treated B16BL6 melanoma cell lines with the EV-inhibitor GW4869 and observed a reduction in cell growth. The authors also confirmed in vivo the efficacy of the use of the inhibitor, showing that the addition of GW4869 was associated with a reduction in the size of the tumor and with an increase in survival time [139]. Another important study focused on how to bypass the resistance after the administration of ICI in melanoma by the use of EV-inhibitors. Given that exosomes expressing PD-L1 may represent a mechanism of resistance, inhibiting the release of these subtypes of exosome could represent a good strategy to improve the efficacy of the treatment. Using an exosome inhibitor (GW4869) and the ferro-apoptosis inducer (Fe^3+^) on B16F10 melanoma cells, the authors showed an increase in the T cells and PD-L1 checkpoint blockade response. In detail, by limiting the uptake of these PD-L1^+^ exosomes by lymph nodes, T cells are free to proliferate and establish a strong anti-tumor effect [140]. To limit the presence of circulating PD-L1^+^ EVs, Davidson et al. recently explored the potential therapeutic use of plasma exchange (TPE); they hypothesize that replacing the plasma of patients with metastatic melanoma may restore the efficacy of PD(L)-1 immunotherapy, by reducing PD-L1^+^-circulating EVs. Even if more pilot studies are needed to assess the safety of TPE, this could be a possible mechanism to reduce EV-mediated drug resistance in melanoma patients treated with ICI [141].

Significantly, it must be considered that EVs are important and play a crucial role not only in tumor development, but also in several other physiological processes. Investigating the different subsets of EVs and being able to act selectively on some of them could represent a strategically successful option with significant future implications.

## 7. Conclusions

Drug resistance is still a big issue in several types of cancer and this is particularly evident in melanoma. Often in this tumor, after an initial response following the administration of the drugs, there is a decrease in the effectiveness and in the response to that drug. Underlying this phenomenon are numerous mechanisms, and a fundamental role is played by EVs. Their contribution in the growth of resistance has been extensively investigated in numerous types of cancers, but few studies have been carried out on melanoma and are mostly descriptive and in vitro only. Summarizing these studies, this review provides a starting point for further investigations focusing on clinical application and highlights the necessity and urgency to acquire a deeper knowledge in order to increase the effectiveness of drugs for the treatment of human melanoma.

## Figures and Tables

**Table 1:** Most frequently used techniques for the isolation of extracellular vesicles [2,7].

Technique	Description
Differential ultracentrifugation	Separates different EVs depending on size and mass. This technique is the most commonly used for the purification of EVs.
Density gradient centrifugation	Use of sucrose or iodixanol density gradient, resulting in a higher level of purity. However, viruses could remain in the final sample.
Precipitation	Final samples obtained are often contaminated by proteins and viruses.
Size Exclusion Chromatography	A very commonly utilized method that isolates EVs depending on size.
Filtration	Separates EVs by passing them through a filter.
Immunoaffinity isolation	Isolates different EVs depending on their surface antigens, with the use of antibodies.
